# Supplementary material for: Meta-Analysis and Gene Set Enrichment Relative to ER Status Reveal Elevated Activity of MYC and E2F in the “Basal” Breast Cancer Subgroup
Source: PLoS One. 2009 Mar 9;4(3):e4710. doi: 10.1371/journal.pone.0004710 (PMC2650420; doi:10.1371/journal.pone.0004710)
Supplement: Dataset S1 — GSEA results from first screen. The file contains a zipped archive of the HTML results of the second GSEA screen. Reports for enriched or depleted datasets may be accessed from the “index.html” link within the my_analysis.GseaPreranked.1219975950625.rpt.zip ZIP archive. Enrichment plots are available for each of the 50 most enriched and depleted gene sets. (13.32 MB ZIP) [file pone.0004710.s001.zip › my_analysis.GseaPreranked.1219975950625.rpt.zip!//BRENTANI_TRANSPORT_OF_VESICLES.html]

Details for gene set BRENTANI\_TRANSPORT\_OF\_VESICLES[GSEA]

|  || Dataset | zSco.ERneg.to.ERpos\_collapsed |
| Phenotype | NoPhenotypeAvailable |
| Upregulated in class | na\_neg |
| GeneSet | BRENTANI\_TRANSPORT\_OF\_VESICLES |
| Enrichment Score (ES) | -0.48310265 |
| Normalized Enrichment Score (NES) | -1.6274431 |
| Nominal p-value | 0.01604278 |
| FDR q-value | 0.10752264 |
| FWER p-Value | 0.998 |
Table: GSEA Results Summary

  

Fig 1: Enrichment plot: BRENTANI\_TRANSPORT\_OF\_VESICLES      
 Profile of the Running ES Score & Positions of GeneSet Members on the Rank Ordered List

  

| PROBE | GENE SYMBOL | GENE\_TITLE | RANK IN GENE LIST | RANK METRIC SCORE | RUNNING ES | CORE ENRICHMENT || 1 | RAB27A |  |  | 1438 | 3.011 | -0.0272 | No |
| 2 | RAB7L1 |  |  | 2511 | 2.194 | -0.0489 | No |
| 3 | RAB33A |  |  | 3921 | 1.545 | -0.1133 | No |
| 4 | RAB2 |  |  | 4263 | 1.409 | -0.1011 | No |
| 5 | RAB5A |  |  | 4447 | 1.336 | -0.0790 | No |
| 6 | AP2M1 |  |  | 5034 | 1.142 | -0.0924 | No |
| 7 | AP3D1 |  |  | 6029 | 0.800 | -0.1456 | No |
| 8 | RAB6A |  |  | 6865 | 0.529 | -0.1942 | No |
| 9 | RAB7 |  |  | 7176 | 0.426 | -0.2061 | No |
| 10 | RAB11A |  |  | 7295 | 0.384 | -0.2046 | No |
| 11 | PIK3C3 |  |  | 7580 | 0.278 | -0.2185 | No |
| 12 | RAB1A |  |  | 8486 | -0.071 | -0.2846 | No |
| 13 | ACTR1A |  |  | 8505 | -0.079 | -0.2839 | No |
| 14 | RAB3B |  |  | 8594 | -0.126 | -0.2871 | No |
| 15 | GOLGA4 |  |  | 9615 | -0.626 | -0.3470 | No |
| 16 | RAB13 |  |  | 10112 | -0.912 | -0.3598 | No |
| 17 | RAB4A |  |  | 10212 | -0.964 | -0.3413 | No |
| 18 | RAB3A |  |  | 10391 | -1.087 | -0.3255 | No |
| 19 | RAB31 |  |  | 12488 | -3.244 | -0.3959 | Yes |
| 20 | RAB27B |  |  | 12576 | -3.378 | -0.3117 | Yes |
| 21 | RAB5C |  |  | 12658 | -3.537 | -0.2228 | Yes |
| 22 | RAB26 |  |  | 12975 | -4.402 | -0.1283 | Yes |
| 23 | RAB5B |  |  | 13237 | -5.738 | 0.0062 | Yes |
Table: GSEA details [plain text format]

  

Fig 2: BRENTANI\_TRANSPORT\_OF\_VESICLES: Random ES distribution      
 Gene set null distribution of ES for **BRENTANI\_TRANSPORT\_OF\_VESICLES**
